# Combination Therapy of Mesenchymal Stromal Cells and Interleukin-4 Attenuates Rheumatoid Arthritis in a Collagen-Induced Murine Model

**DOI:** 10.3390/cells8080823

**Published:** 2019-08-03

**Authors:** Shaimaa M. Haikal, Nourtan F. Abdeltawab, Laila A. Rashed, Tarek I. Abd El-Galil, Heba A. Elmalt, Magdy A. Amin

**Affiliations:** 1 Department of Microbiology and Immunology, Faculty of Pharmacy, Cairo University, Cairo 11562, Egypt; 2 Department of Biochemistry and Molecular Biology, Faculty of Medicine, Cairo University, Cairo 11562, Egypt; 3 Department of Anatomy and Embryology, Faculty of Medicine, Cairo University, Cairo 11562, Egypt; 4 Department of Medical Biochemistry, National Research Center, Cairo 11562, Egypt

**Keywords:** rheumatoid arthritis, auto-inflammatory disease, cytokines, IL-4, mesenchymal stem cells, mouse model, collagen-induced arthritis, inflammatory mediators

## Abstract

Rheumatoid arthritis (RA) is a disease of the joints that causes decreased quality of life. Mesenchymal stromal cells (MSCs) have immunosuppressive properties, with possible use in the treatment of RA. Similarly, interleukin (IL)-4 has been shown as a potential RA treatment. However, their combination has not been explored before. Therefore, this study aimed to investigate the effect of a combination therapy of MSCs and IL-4 in the treatment of RA, using a murine collagen-induced arthritis (CIA) model. Arthritis was induced in Balb/c mice by two intradermal injections of type II collagen (CII), at days 0 and 21. CIA mice were randomly assigned to four groups; group I received an intravenous injection of mouse bone marrow-derived MSCs, while group II received an intraperitoneal injection of IL-4. Group III received a combined treatment of MSC and IL-4, while group IV served as a CIA diseased control group receiving phosphate buffer saline (PBS). A fifth group of healthy mice served as the normal healthy control. To assess changes induced by different treatments, levels of RA-associated inflammatory cytokines and biomarkers were measured in the serum, knee joints, and synovial tissue, using ELISA and Real Time-qPCR. Histopathological features of knee joints were analyzed for all groups. Results showed that combined MSC and IL-4 treatment alleviated signs of synovitis in CIA mice, reverting to the values of healthy controls. This was evident by the decrease in the levels of rheumatic factor (RF), C-reactive protein (CRP) and anti-nuclear antibodies (ANA) by 64, 80, and 71%, respectively, compared to the diseased group. Moreover, tumor necrosis factor-alpha (TNF- α) and monocyte chemoattractant protein-1 (MCP-1) levels decreased by 63 and 68%, respectively. Similarly, our gene expression data showed improvement in mice receiving combined therapy compared to other groups receiving single treatment, where cartilage oligomeric matrix protein (Comp), tissue inhibitor metalloproteinase-1 (Timp1), matrix metalloproteinase1 (Mmp-1), and IL-1 receptor (Il-1r) gene expression levels decreased by 75, 70, and 78%, respectively. Collectively, treatment with a combined therapy of MSC and IL-4 might have an efficient therapeutic effect on arthritis. Thus, further studies are needed to assess the potential of different MSC populations in conjugation with IL-4 in the treatment of experimental arthritis.

## 1. Introduction

Rheumatoid arthritis (RA) is characterized by inflamed joints causing pain, swelling and stiffness. It is an autoimmune disease mediated by autoreactive T cells entering the joint tissues and releasing pro-inflammatory cytokines. Inflammatory cytokines promote macrophage and neutrophil infiltration and activation in joint tissues [1]. It has been found that enhancing the frequency and immunosuppressive function of regulatory T cells (Treg) may provide an important therapeutic strategy for RA [2]. RA is one of the most common autoimmune joint diseases and has witnessed significant therapeutic advances in the past decade. Although significant progress has been made in RA treatment for obtaining long-term remission-induction, 20–30% of patients with moderate-to-severe RA do not respond positively to mono or combination therapy (plus methotrexate) with these agents. Thus, the development of novel therapies is needed to improve RA progression [3].

Pre-clinical research has demonstrated that adipose, umbilical cord, and bone marrow-derived mesenchymal stromal cells (MSCs) all have the ability to suppress the functions of different immune cells, providing possible therapies for autoimmune diseases including RA [4]. Bone marrow-derived MSCs (BM-MSCs) are multipotent adult stem cells used in organ repair and cell therapy. The application of MSCs for tendon-bone healing is reported with promising results. MSCs can differentiate into osteoblasts, chondrocytes, and adipocytes and are possibly regulated by specific signals under injury conditions. In vivo and in vitro studies showed the potential of MSCs to differentiate into osteoblasts. Cytokines involved in cell migration can direct MSCs to the injured sites, where they play a significant role in tissue regeneration [5]. It has been suggested that MSCs can restore the balance between pro-inflammatory and anti-inflammatory memory T cell populations deregulated in RA, which prompts their therapeutic function [3]. Moreover, MSCs’ immunomodulation is partially due to the secretion of various immunoregulatory factors, including IL-4, IL-10, and transforming growth factor-beta (TGF-β), reviewed in Ben-Ami E. et al. (2011) and Kyurkchiev D. et al. (2014) [6,7].

IL-4 is a pleiotropic cytokine and is considered as a promising modulator in the control of RA. IL-4 was found useful in the treatment of various autoimmune models in vivo, including CIA and proteoglycan-induced arthritis (PGIA) [8,9]. IL-4 has been shown to prevent joint damage and bone erosion, to ameliorate arthritis, and to promote tissue repair in CIA mouse models [10,11,12]. Since IL-4 acts as an anti-inflammatory cytokine, it modulates macrophage polarization and activity through the suppression of Th1-mediated pro-inflammatory effects, thus enhancing Th2-mediated anti-inflammatory effects, possibly via the modulation of histone deacetylation [13].

Therefore, the current study aimed to investigate the effect of combining BM-MSCs and IL-4 in the treatment of RA using a murine model of CIA. We found that using MSCs and IL-4 in combination had the most significant effect in reducing RA, studied as CIA in a murine model.

## 2. Materials and Methods

### 2.1. Animals

A total of 60 Balb/c male mice (6–8 weeks old) were used in this study. Mice had access to food and water ad libitum and were housed in an environmentally controlled room with 12 h light/dark periods. All animal experiments were conducted in agreement with the institutional regulations. The Research Ethics Committee of the Faculty of Pharmacy, Cairo University, Egypt approved all animal studies (protocol approval number MI (956)).

### 2.2. Collagen-Induced Arthritis Model

Four groups of male Balb/c mice (n = 40) received 100 μg of bovine type II collagen (CII) emulsified in complete Freund´s adjuvant (CFA) intradermally at the base of the tail, at day 0 then at day 21 as an immunization boost dose, following an established procedure [14]. After immunization until the end of experimental period, mice were examined daily for the onset of clinical manifestations of arthritis. Some mice had severe disease and succumbed to the disease prior to receiving any treatment, while the rest of the animals had intermediate severity, which was apparent starting at 15 days from the first immunization, similar to what was previously published by others and our co-authors [15,16]. Moreover, disease development and MSC alleviation of disease severity during the current study was closely similar to our previous study, El-denshary, ESM. et al. (2014) [16], and histopathological examination showed severe, moderate and mild CIA (Supplementary Figure S1).

### 2.3. Preparation of BM-Derived MSCs

Bone marrow was harvested by flushing the tibiae and femurs of ten healthy male Balb/c mice with Dulbecco’s modified Eagle’s medium (DMEM) (Gibco, Life Technologies Ltd., Paisley, Scotland, UK), supplemented with 10% fetal bovine serum (Gibco, Life Technologies Ltd., Paisley, Scotland, UK). Nucleated cells were isolated with a density gradient Ficoll/Paque (GE Healthcare Life Sciences, Buckinghamshire, UK) and resuspended in complete culture medium supplemented with 1% penicillin–streptomycin (Gibco, Life Technologies Ltd., Paisley, Scotland, UK). Cells were incubated at 37 °C in 5% humidified CO_2_ for 12–14 days as a primary culture. When large colonies developed and reached ~70–80% confluence, cultures were washed twice with phosphate buffer saline (PBS). Cells were then trypsinized with 0.25% trypsin in 1 mM Ethylenediaminetetraacetic acid (EDTA) (Gibco, Life Technologies Ltd., Paisley, Scotland, UK) for 5 min at 37 °C. After centrifugation, cells were resuspended in serum-supplemented medium and incubated in a 50 cm^2^ culture flask. The resulting cultures were referred to as first-passage cultures. As is characteristic of MSCs, cells examined under the microscope were adhesive and had fusiform shape. We detected CD29 and CD105 by flow cytometry as surface markers of MSCs, as previously described [17].

### 2.4. Experimental Design

**Figure 1:**
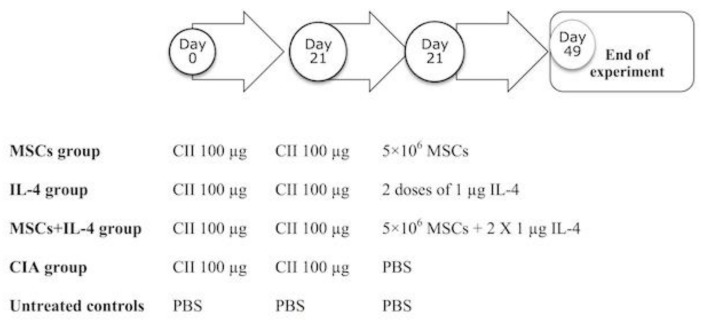
Illustration of the general scheme of the experimental design of this study. Rheumatoid arthritis (RA) was induced using a collagen-induced arthritis (CIA) murine model in four groups of mice (n = 10 each). A fifth healthy control group (n = 10) was also included for comparison. The schedule of administration of type II collagen (CII) injection and treatments are illustrated. Mesenchymal stromal cells (MSCs), interleukin (IL)-4, and phosphate buffer saline (PBS).

We induced arthritis in four groups of mice as detailed above using CII (Figure 1). The first group of mice, the MSC group (n = 10), received MSCs by treating CII-immunized Balb/c mice with an intravenous (i.v.) injection cell suspension containing 5 × 10^6^ allogeneic MSCs, at day 21 [18]. The IL-4 group (n = 10) received two doses of 1 µg of IL4 at the moment of the boost at day 21 [19]. The MSC + IL-4 group (n = 10) received 5 × 10^6^ MSCs and two doses of 1 µg of IL-4 at the moment of the boost at day 21. CIA group mice received 1 mL PBS by tail vein injection [20] (Figure 1). Finally, we had a fifth group of healthy untreated mice. After four weeks of the various treatments, mice were sacrificed, and blood and synovial tissues collected. Sera were isolated and used for the determination of levels of rheumatic factor (RF), C-reactive protein (CRP) and anti-nuclear antibodies (ANA), using ELISA. Synovial tissues were examined for IL-10, TNF-alpha and MCP-1 levels and differential gene expression of Comp, Mmp-1, Timp-1 and Il-1r.

### 2.5. Biochemical Analysis of Rheumatoid Markers

Serum RF was detected by a QUANTA Lite ELISA kit (Inova Diagnostics, San Diego, CA, USA), and serum levels of CRP and ANA were assayed using ELISA (DRG Diagnostics, Marburg, Germany), following the manufacturer’s protocol. IL-10, TNF-alpha, and MCP-1 cytokine levels were detected by an ELISA kit (Wkea Med supplies, Changchun, China), following the manufacturer’s instructions.

### 2.6. RNA Isolation and Reverse Transcription 

RNA was extracted from synovial tissue homogenate by a Total RNA purification kit, (Jena Bioscience, Jena, Germany), according to the manufacturer’s instructions. RNA was subsequently used for cDNA synthesis using a reverse transcription system cDNA synthesis kit (iNtron Biotechnology, Seongnam, South Korea), following the manufacturer’s protocol.

### 2.7. Quantitative Real-Time PCR

We used SYBR-GREEN chemistry real-time PCR (BIORON, Ludwigshafen, Germany) for gene expression analysis on a StepOnePlus™ Real-Time PCR System (Applied Biosystems, Foster City, California, USA). We used cycling conditions as follows: 10 min at 95 °C, followed by 40 cycles of 15 s at 95 °C and 60 s at 60 °C. Primer pair sequences used are shown in Table 1. Data were analyzed with the ABI 7500 Prism system software (Applied Biosystems, Foster City, California, USA). The relative expression of studied genes was calculated using the delta-delta Ct method (∆∆Ct). All values were normalized to beta-actin gene, used as the housekeeping gene [21].

### 2.8. Analysis of Knee Joint Histopathology

Joint tissue sections were collected on glass slides, which were then deparaffinized and stained with hematoxylin and eosin. Slides were examined using an electric microscope with a magnification power of ×200. An arthritis semi-quantitative score was calculated based on Simon et al. (2001) [22]. Briefly, inflammation was assessed as synovial lining layer hyperplasia and infiltration of leukocytes into synovial membrane/joint space. Tissue destruction was assessed from pannus formation and necrosis/erosion of cartilage. The final arthritis score is the sum of the values for inflammation and destruction.

### 2.9. Statistical Analysis

Significant differences between treatments were determined using One-way ANOVA, Kruskal–Wallis test and Dunn’s multiple comparisons. All statistical analyses were performed using GraphPad Prism 6.01 (GraphPad Software Inc., San Diego, CA, USA). P values less than 0.05 were considered significant.

## 3. Results

### 3.1. Combined MSCs and IL-4 Treatment Showed Best Improvement in Biochemical Markers of RA

**Figure 2:**
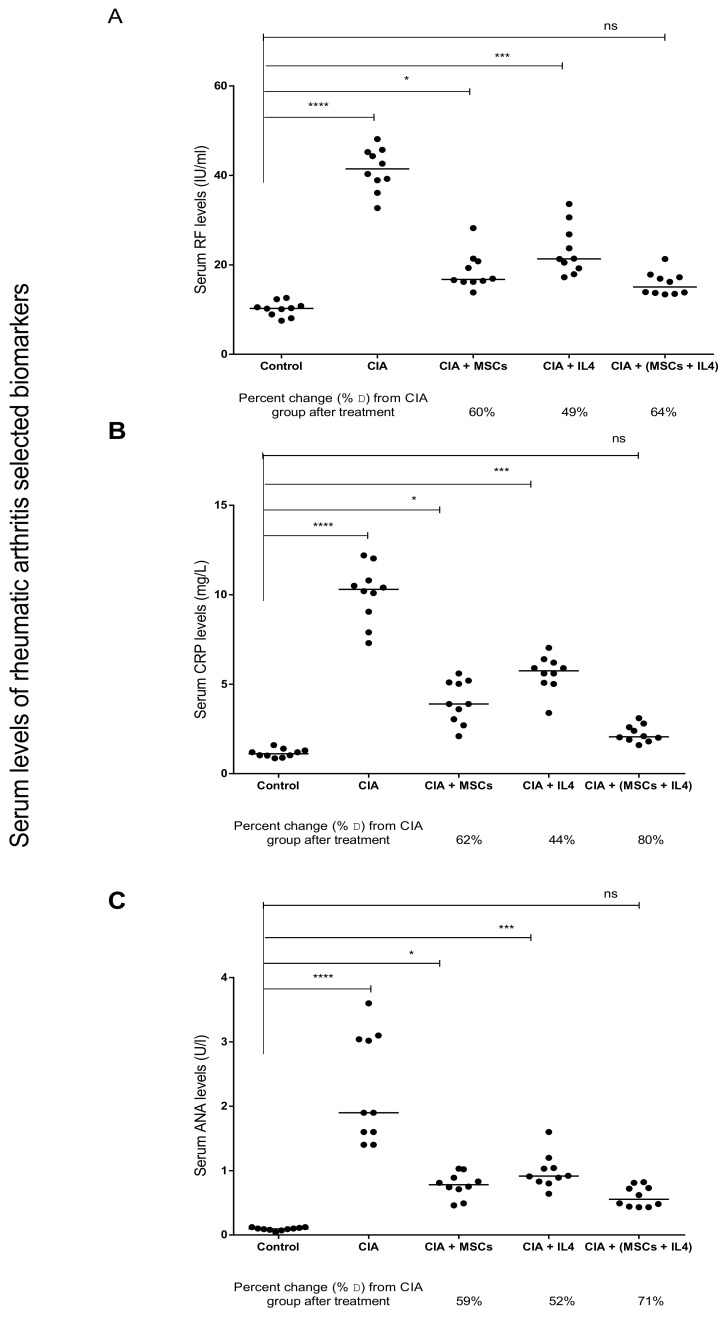
MSCs and IL-4 combined therapy attenuates arthritis in a CIA murine model. Comparison of serum levels of arthritis associated biomarkers in mice treated with CII compared to healthy control group. CIA mice treated with MSCs and IL-4, alone or combined, showed attenuation of CIA, with a varying degree of significance. (**A**) Rheumatic factor (RF) serum levels; (**B**) C-reactive protein (CRP) serum levels; and (**C**) anti-nuclear antibody (ANA) serum levels. Medians were compared using One-way ANOVA, Kruskal–Wallis test and Dunn’s multiple comparison test. *P* value <0.05 was considered significant. (MSCs: mesenchymal stem cells, IL-4: interleukin-4, CIA: collagen-induced arthritis, CII: type II collagen)

The induction of RA was successful, as indicated by a significant increase in serum levels of RF, CRP and ANA in the CIA group in comparison to the healthy control group (Figure 2). When either MSCs or IL-4 were used as therapy, there was a significant decrease in the RF serum levels compared to the CIA group, by 60% and 49%, respectively (Figure 2A). On using combined MSCs and IL-4 as a treatment, RF levels in the blood significantly decreased by 64% compared to the CIA group (Figure 2A). Similar results were observed with CRP serum levels; when MSCs or IL-4 were injected into the mice with induced RA, there was a significant decrease in the serum CRP concentration compared to the CIA group, by 62% and 44%, respectively (Figure 2B). After using combined treatment, we found a marked significant decrease in CRP serum levels compared to the CIA group, by 80%, returning to the levels of the healthy control group (Figure 2B). As for ANA, treatment with MSC or IL-4 showed a significant decrease in ANA serum levels, by 59 and 52%, respectively, compared to the CIA group. Using combined treatment led to a significant drop in serum ANA levels, by 71%, compared to the CIA group, and ANA levels almost reached the same average level as the control group (Figure 2C). Collectively, serum biochemical hallmarks of RA decreased significantly in combined therapy using MSCs and IL-4, reaching the levels of healthy controls (Figure 2).

### 3.2. Combined MSCs and IL-4 Attenuated RA via Anti-Inflammatory Action

**Figure 3:**
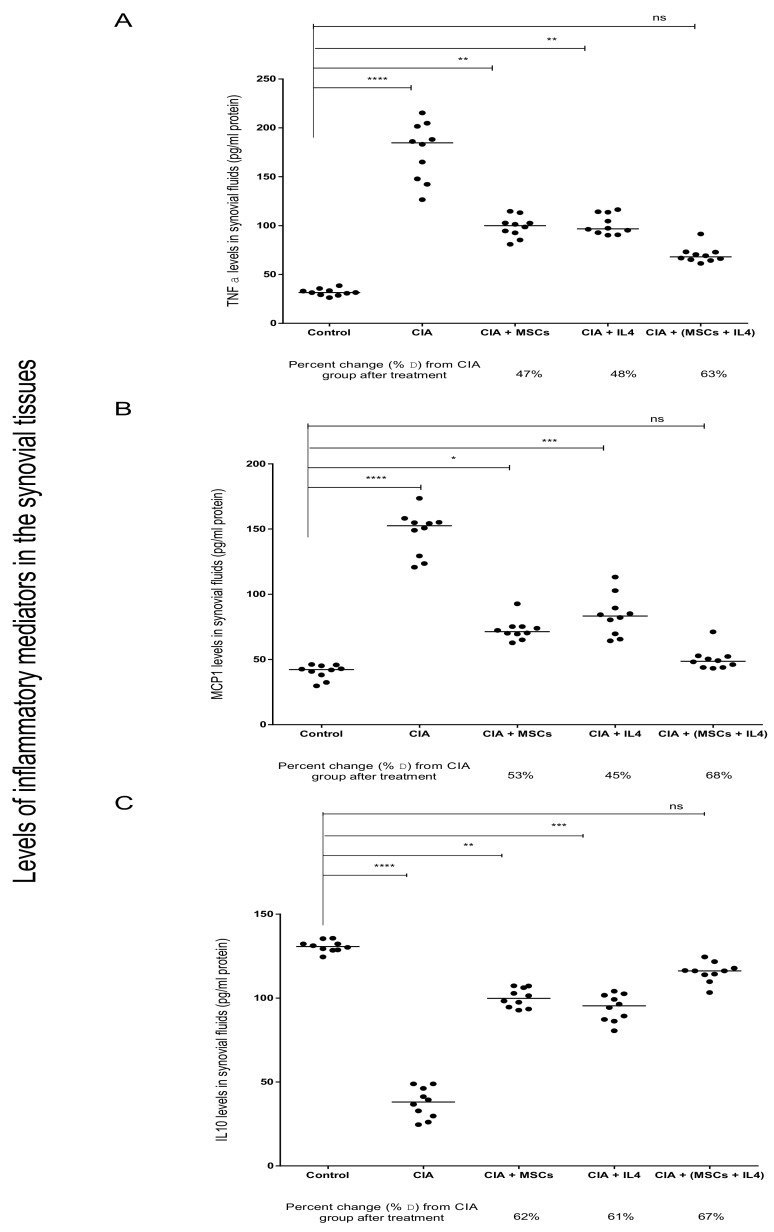
Decrease in pro-inflammatory and increase in anti-inflammatory cytokine levels in synovial tissue, in CIA mice treated with double therapy of MSCs and IL-4. Comparison of pro-inflammatory (**A**) TNF-α and (**B**) MCP-1 levels in synovial tissues, in mice treated with CII, compared to the healthy control group. CIA mice treated with MSCs and IL-4, alone or combined, showed attenuation of CIA, where TNF- α and MCP-1 decreased following treatment, with a varying degree of significance. Meanwhile, anti-inflammatory (**C**) IL-10 levels in synovial tissues of CIA mice treated with MSCs and IL-4, alone or combined, showed an increase in levels and attenuation of CIA. Medians were compared using One-way ANOVA, Kruskal–Wallis test and Dunn’s multiple comparison test. P value <0.05 was considered significant.

After the induction of arthritis in mice, TNF-α and MCP-1 levels increased significantly while IL-10 levels decreased significantly in the synovial tissues of the CIA group, compared to the healthy control group (Figure 3). After using either MSC or IL-4 as a treatment, we found a significant improvement in TNF-α synovial levels, by 47% and 48%, compared to the CIA group (Figure 3A). Treatment with the combination of MSCs and IL-4 showed a marked improvement in TNF-α levels compared to the diseased levels, by 63% (Figure 3A). MCP-1 levels decreased significantly after treatment with either MSC or IL-4, compared to the CIA group, by 53% and 45%, respectively (Figure 3B). Finally, on using MSCs and IL-4 as a combined treatment, we found a significant improvement in MCP-1 levels, by 68%, compared to the CIA group, with MCP-1 levels almost reaching the same average level as the control group (Figure 3B). The anti-inflammatory cytokine IL-10 levels increased significantly after using MSCs or IL-4 as a treatment, by 62% and 61%, respectively, compared to the CIA group (Figure 3C). After using combined treatment, IL-10 levels increased by 67% compared to the CIA group (Figure 3C).

### 3.3. Combined MSCs and IL-4 Attenuated Articular Cartilage Degradation and Positively Affected Connective Tissue Remodeling of Synovial Tissue

**Figure 4:**
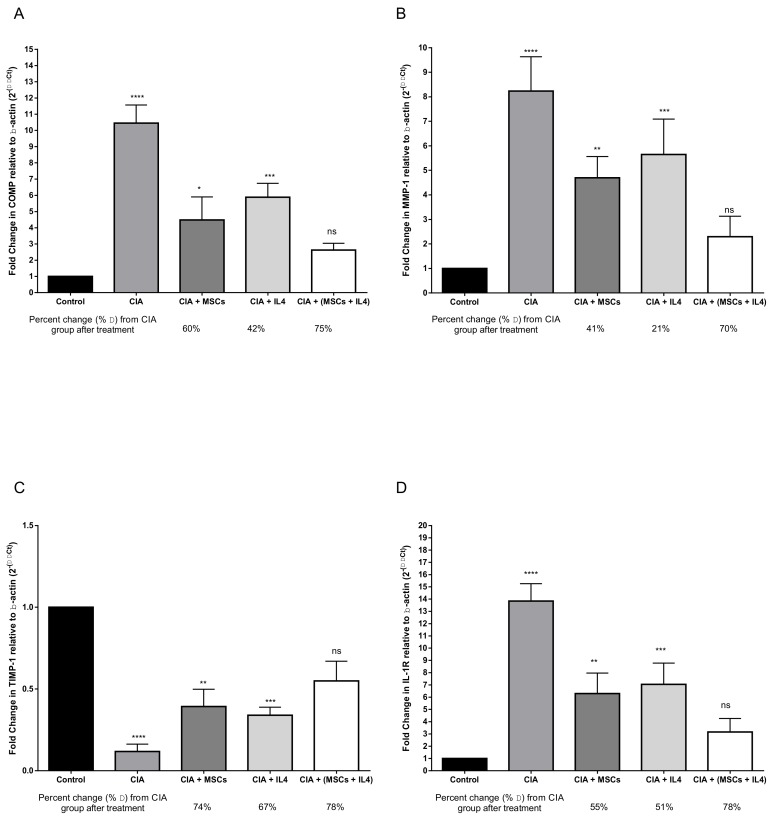
Differential gene expression analysis of COMP, MMP-1, TIMP-1 and IL-1R in synovial tissues of CIA mice treated with MSCs, IL4 or combined therapy. Comparison of (**A**) COMP, (**B**) MMP-1, (**C**) TIMP-1 and (**D**) IL-1R levels in mice treated with CII compared to the healthy control group. CIA mice treated with MSCs and IL-4, alone or combined, showed attenuation of CIA, with a varying degree of significance. Means ± SD were compared using One-way ANOVA, Kruskal–Wallis test and Dunn’s multiple comparison test. *P* value <0.05 was considered significant.

Next, we aimed to examine the effect of treatments on joints, along with their cartilage and synovial fluid, by studying the differential expression of synovial and cartilage markers. The CIA group showed a significant increase in COMP, MMP, and IL-1R gene expression levels coupled with a decrease in TIMP-1 gene expression, compared to the healthy control group. Upon treatment with MSCs, a significant decrease in COMP levels was observed, compared to the diseased group, by 60% (Figure 4A). After treatment with IL-4, there was a significant reduction in COMP gene expression, by 42%, compared to the CIA group. When combined treatment was used, COMP gene expression significantly decreased compared to the CIA group, by 75% (Figure 4A). After either MSC or IL-4 treatment, MMP-I gene expression significantly decreased compared to the CIA group, by 41% and 21%, respectively (Figure 4B). In the combined treatment group, MMP-1 gene expression levels decreased by 70% compared to CIA group and almost reached healthy control group levels (Figure 4B). Gene expression of IL-1R type 1 significantly decreased after using MSCs as a treatment, compared to CIA group, by 55% (Figure 4C). As for IL-4’s effect, IL-1R gene expression levels decreased significantly, by 51%, compared to the diseased group (Figure 4C). After using combined treatment, there was a significant improvement in IL-1R gene expression levels, by 78%, compared to the CIA group, which was better than each treatment alone (Figure 4C). TIMP-1 gene expression levels were increased significantly after injection of either MSCs or IL-4 into mice as a treatment, by 74% and 67%, respectively, compared to the CIA group (Figure 4D). The result of using a combined treatment showed a significant increase in TIMP-1 gene expression levels compared to the CIA group, by 78% (Figure 4D).

### 3.4. Combined MSCs and IL-4 Improve the Histopathological Changes in the Knee Joint

**Figure 5:**
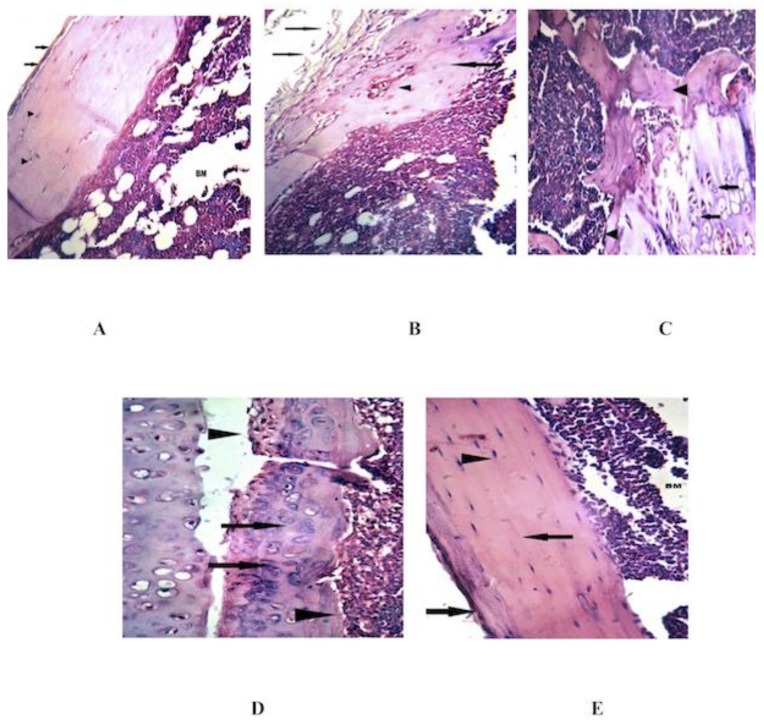
Comparison of histopathology of knee joint synovial membranes of control, CIA mice and treatment groups. Shown are representative Hematoxylin and eosin (H&E) stained sections of the knee joint, examined by light microscopy from (**A**) control and (**B**) CIA mice with erosion of articular cartilage, edema, and a massive influx of inflammatory cells into the subsynovial connective tissue. (**C**) MSC treated mice showing migration of stem cells and regeneration of synovial membrane, with absence of inflammatory cell infiltrate and exudates. (**D**) IL-4 treated joint showed regeneration of synovial membrane cells, near normal bone structure, mild inflammatory cell infiltrate, no edema nor exudates. (**E**) Joint treated by combined treatment showed intact synovial membrane, regenerated normal synovial tissue and normal bone marrow.

Histologic analysis of knee joint sections of the RA group revealed severe destruction of the synovial membrane, hyperplasia of the synovial membrane, edema, exudates and a massive influx of inflammatory cells into the sub synovial connective tissue (Figure 5B). After treatment with MSCs, the knee joint sections showed regeneration of synovial membrane cells, healthy articular cartilage, normal synovial membrane, no inflammatory cell infiltrate, no edema and no exudates (Figure 5C). The knee joint sections of mice treated with IL-4 showed regeneration of synovial membrane cells, healthy articular cartilage, mild inflammatory cell infiltrates, no edema, no exudates and no proliferation of synovial membrane (Figure 5D). In the combined treatment, the knee joint sections showed normal intact synovial membrane, regenerated normal synovial tissues, healthy articular cartilage, normal bone marrow, no inflammatory cell infiltrate, no edema, no exudates and no erosion of articular cartilage (Figure 5E). Only IL-4 alone or combined MSCs and IL-4 returned the diseased group to regular status, like the control. Table 2 shows a semi-quantitative evaluation of histopathological changes and an arthritis score based on Simon et al., 2001.

## 4. Discussion

The exact causes and disease mechanism of RA are not yet fully understood; however, the destructive lesions of RA are known to result from immune inflammatory processes [23]. To better understand RA, several animal models of arthritis have been used [24]. Although animal models cannot replicate the exact clinical manifestations of RA, several mouse models have been used to understand possible treatments for RA. One of the established mouse RA models is the CIA model, which ensures that some of the clinical, histopathological and immunological features are similar to human RA [25]. In the current study, CIA in mice showed a similar pathology to human RA and thus was selected to try to predict the efficacy of the proposed combined therapy in humans [26,27]. Several mouse strains have been used previously in established CIA mouse models for chronic RA, including Balb/c mice [27]; therefore, we used this model for testing our proposed combined treatment of RA using MSCs and IL-4.

Several studies indicated that MSCs possess an immunosuppressive role, with potential in the treatment of RA [28,29,30,31,32]. Furthermore, IL-4, an anti-inflammatory cytokine, has been reported to attenuate RA. This is attributed to IL-4 balancing the abnormal activity of Th1 cells and monocytic cell activation [33]. Therefore, we aimed to harness the benefits of combining the immunosuppressive reparative potentialsof MSCs and the anti-inflammatory actions of IL-4 in an RA animal model. We followed previously established protocols for the use of MSCs [18] and IL-4 [19] in preclinical RA therapy. Lower doses of IL-4 can be used for a longer time, according to previous studies. However, during our standardization, we found that a higher and less frequent IL-4 dose, as indicated in the experimental section, was the most effective dose. The effect might be due to the anti-inflammatory effect of IL-4 exerted at an early stage of arthritis development. We assessed the potential efficacy of a combined therapy of MSC and IL-4 by measuring different inflammatory markers in blood and synovial fluid, and by histological examination of joint tissue. MSCs homing to the joint tissue was carried out previously, where MSCs were injected into the same CIA model [16]. Meanwhile, another method of treatment was used in El-Dinshary et al. (2014) [16], in which MSCs labeled with PKH26 fluorescent dye were detected in the joint knee tissue. Injected MSCs were labeled with PKH26 and showed strong red auto-fluorescence after transplantation into mice, confirming that these cells were seeded into the joint knee tissue [16].

Treatment of CIA mice with combined MSC and IL-4 showed a beneficial effect, reducing RF, CRP, and ANA levels compared to MSCs or IL-4 alone. Serum RF antibodies are commonly regarded as the serological hallmark of RA; however, they may be present in a variety of other rheumatic and non-rheumatic conditions, and also among healthy individuals [34]. Treatment with IL-4 or MSCs alone has been previously shown to decrease RF levels in a mouse model of RA [16]. CRP is a prototypical acute phase protein in humans and an important mediator of host defense. Serum levels of CRP have been used as a prognosis for progressive joint damage and functional status and outcome [35]. Loyer et al. (1993) [36] reported that IL4 can decrease CRP production, and they considered IL-4 as a modulator of hepatic plasma protein production and supported the idea of its anti-inflammatory function. ANA, a specific class of autoantibodies, have the capability to bind and destroy certain structures within the nuclei of cells. Although lower amounts of these antibodies can be seen in the normal population, high titers are seen in connective tissue disease (CTD). ANA are involved in the disease pathogenesis and also constitute the basis for diagnosis and treatment of CTD [37]. After injection of MSCs, we found a significant decrease in ANA serum levels in the MSC treated group, compared to the RA group, in agreement with [38]. There are several hypotheses regarding mechanisms by which autoantibodies might lead to RA symptoms, as reviewed in Derksen V et al. (2017) [39]. Evidence suggests that autoantibodies have the potential to augment the immune response in RA by both Fcγ receptor binding and complement activation. Moreover, the augmented generation of neutrophil extracellular traps (NETs) is another manner in which anti-citrullinated peptide/protein antibodies (ACPA) and/or RF antibodies could affect disease development or persistence [40]. In addition, it has been proposed that autoantibodies may directly affect osteoclasts and thereby lead to the formation of bone erosions [41,42].

The differential gene expression of synovial and cartilage markers (COMP, MMP, TIMP and IL-1R) showed significant improvement by using combined MSCs and IL-4 therapy. COMP, a prominent constituent of articular cartilage, has been reported to increase in patients with knee osteoarthritis (OA) and early RA [43]. It has been proposed that COMP molecules are important for maintaining the properties and integrity of the collagen network and contribute to the material properties of biological tissue [44]. MSCs alone caused a significant decrease in COMP levels compared to the diseased group, in agreement with [16] who measured COMP gene expression in mice after using MSCs as a treatment. After treatment with IL-4, there was also a significant decrease in COMP gene expression compared to the RA group, in agreement with Joosten et al., 1999 [19]. When combined treatment was used, a significant decrease in COMP expression levels compared to the RA group was observed, better than each treatment alone.

MMP family members are the major enzymes that degrade the components of the extracellular matrix. Expression of MMPs is low in normal cells, and these low levels allow for healthy connective tissue remodeling. In pathologic conditions, the level of MMP expression increases considerably, resulting in aberrant connective tissue destruction. Excess MMP production is associated with the pathology of many diseases, including arthritic disease [45]. After the induction of arthritis, MMP-1 gene expression was significantly increased compared to the healthy control group, in agreement with [46]. Using MSCs, we found a significant decrease in MMP-1 gene expression, similar to Kim et al., 2007 [47], who investigated possible roles of adipose-derived stem cells (ADSCs) in the skin wound healing process. IL-4 treatment significantly decreased MMP-1 gene expression levels compared to the RA group, in conformity with [48] who determined the effects of Th2-type cytokines (interleukin (IL)-4 and -13) on conjunctival fibroblasts of vernal keratoconjunctivitis (VKC). Using combined treatment, our results showed further improvement compared with each treatment alone, where MMP-1 gene expression levels almost reached the healthy control group levels.

The activity of MMPs is regulated by their endogenous inhibitors, tissue inhibitors of metalloproteinases (TIMPs) [49]. TIMPs regulate the breakdown of extracellular matrix components and play an important role in tissue remodeling and growth, in both physiological and pathological conditions [50]. In our study, TIMP-1 gene expression levels were decreased significantly in the RA group compared to the healthy control group, in agreement with [51] who measured TIMP-1 in synovial fluid (SF) aspirated from knee joints of 97 patients. Yoshihara et al. (2000) [51] found that the levels of TIMP-1 in SF in the different RA stages when compared were high, even in the early stage, and decreased significantly in the advanced stage. After injection of MSCs into mice as a treatment, we found a significant increase in TIMP-1 expression compared to the RA group, in agreement with [52]. When using IL-4 as an injection in a CIA model, the TIMP-1 gene expression level increased significantly compared to the diseased group, in concordance with [48] where an increase in the expression of the TIMP-1 gene was induced by 10 ng/mL of IL-4. The result of using a combined treatment showed a significant increase in TIMP-1 gene expression levels compared to the RA group. Combined treatment revealed a great improvement in TIMP-1 levels compared to its levels after using IL-4 treatment alone.

Another important biomarker of RA is IL-1R, a cytokine receptor which binds interleukin-1. Two forms of the receptor exist: the type I receptor is primarily responsible for transmitting the inflammatory effects of interleukin-1 (IL-1), while type II receptors may act as a suppressor of IL-1 activity by competing for IL-1 binding [53]. Also opposing the effects of IL-1 is the IL-1 receptor antagonist (IL-1RA) [54]. In our study, we measured the gene expression of IL-1R type 1, which is also known as CD121a, in synovial tissue. We found a significant increase in IL-1R expression levels in the RA group compared to the control group. When MSCs were used as a treatment, IL-1R gene expression significantly decreased compared to the RA group, in agreement with [1] who found a significant decrease in IL-1β protein expression in joint extracts from CIA mice treated with human adipose-derived (AD) MSCs, suggesting that a decrease in the agonist IL-1β and an increase in the antagonist IL-1Ra may lead to a decrease in the level of IL-R, which is responsible for transmitting the inflammatory effects of interleukin-1 [1,55]. As for IL-4’s effects on IL-1R, [56] measured the IL-1β level in ankle homogenate in an adjuvant-induced arthritic rat model, after a single injection of adenovirus as a source of rat IL-4. They found that the ankle homogenate levels of IL-1β were decreased by 76%. [57] studied the effect of exogenous IL-4 on IL-1 Ra and IL- lβ production, when added to the culture of rheumatoid synovium pieces. They found that IL-4 significantly increased the levels of IL- lRa while reducing IL-lβ levels. Thus, IL-4 may be of therapeutic interest in RA. The results reported by these two studies support our results as after using IL-4 as a treatment, IL-1R gene expression levels decreased significantly compared to the diseased group. Moreover, after using combined treatment, there was a significant improvement in IL-1R gene expression levels compared to the use of each treatment alone.

IIL-10 has indispensable functions in inflammatory diseases such as RA [58] as it is essential for maintaining the integrity and homeostasis of tissue epithelial layers. It can facilitate the tissue-healing process in injuries caused by infection or inflammation as it represses pro-inflammatory responses. In addition, it limits unnecessary tissue disruptions caused by inflammation. IL-10 levels in the synovial tissue of the RA group decreased significantly compared to the healthy control group. A significance increase in IL-10 levels after using MSCs as a treatment was found, similar to the results reported by [20] who measured the effect of umbilical cord-derived MSCs (UCMSC) in RA. After using IL-4 as a treatment, we found a significant increase in IL-10 levels compared to the RA group. [59] measured IL-10 levels after using IL-4 on lipopolysaccharide (LPS)-treated murine peritoneal macrophages, in vitro. They found a significant increase in IL-10 release, where IL-10 was a potent deactivating factor produced endogenously by macrophages. IL-10 is part of the negative feedback loop controlling cytokine production. Therefore, the augmentation of IL-10 is likely to be important for the maintenance of the Th1/Th2 balance. Therefore, the modulation of IL-10 by IL-4 may be an important step in the regulation of the immune system in RA. We also measured levels of TNF-α in synovial tissue, which is a pro-inflammatory cytokine produced during acute inflammation and is responsible for a diverse range of signaling events within cells [60]. After the induction of arthritis, TNF-α levels increased significantly in synovial tissues of the RA group compared to the healthy control group, in agreement with [14]. After using IL-4 as a treatment, we found a significant improvement in TNF-α synovial levels, as seen in [56]. Treatment with the combination of MSCs and IL-4 showed a significant improvement in TNF-α levels compared to the diseased group and even when using each treatment alone. Finally, to evaluate the induction of arthritis in mice, MCP-1 levels in synovial tissue were measured. A significant increase in MCP-1 level was observed in the RA group in comparison with the control group, as seen in [1], suggesting that stem cells might have decreased protein expression of various inflammatory mediators, including MCP-1. By using MSCs and IL-4 as a combined treatment, we found a significant improvement in MCP-1 levels, much more than using each treatment alone. MCP-1 levels almost reach the same normal level as the control group.

Mice immunized with CII developed polyarthritis, characterized by severe cartilage and bone erosions. In addition to erythema and edema, histopathological features included synovitis, pannus formation, cartilage and bone erosion and a massive influx of inflammatory cells into the subsynovial connective tissue, as previously reported [26,61]. Histological assessment of knee joints after treatment with MSCs alone showed regeneration of synovial membrane cells, normal articular cartilage, and normal synovial membrane, in concordance with previous studies [31,62,63]. The knee joint sections of mice treated with IL-4 showed regeneration of synovial membrane cells, normal articular cartilage, and mild inflammatory cell infiltrate with no proliferation of synovial membrane, in conformity with [63]. Kim et al. (2001) [63] found that joints from dendritic cells (DC)/IL-4-treated mice showed less inflammatory joint tissue, a reduction in bone erosion, and a reduction in the number of inflammatory cells, upon histologic analysis. After treatment with combined MSCs and IL-4, the knee joint sections showed normal intact synovial membranes and regenerated synovial tissues.

## 5. Conclusions

The results of the current study demonstrate that a combined therapy using MSCs and IL-4 reduced joint inflammation, synovial cellularity, levels of pro-inflammatory cytokines, vascularization, and bony destruction in a CIA animal model. In addition, the treatment showed improved biochemical markers in the CIA models, as RF, CRP and ANA significantly decreased. COMP, MMP-1, IL-1R gene expression levels were significantly decreased while TIMP-1 was significantly increased. Finally, anti-inflammatory action was proved by the significantly decreasing levels of TNF-α and MCP-1 and significantly increasing IL-10 levels. Therefore, the use of MSCs combined with IL-4 might have a more efficient therapeutic effect on CIA, with promise for use in humans. These promising results of a combined therapy need further studies to decipher the effective dose, mode of administration, and the detailed mechanisms of protection.

## Figures and Tables

**Table 1:** Primer sequences used for real-time PCR analysis.

Gene	Primer Sequence	
	Forward Primer	Reverse Primer
Comp1	5′- ATGGTCTTACGGGGAGATGCC -3′	5′- GAGAGGTTTCTGGAGCCTTTTGG -3′
Mmp1	5′- TTGTTGCTGCCCATGAGCTT -3′	5′- ACTTTGTCGCCAATTCCAGG -3′
Timp1	5′- GCATCTGGCATCCTCTTGTT-3′	5′- TGGGGAACCCATGAATTTAG-3′
Il1r	5′- TGAGGTCTTGGAGGGACAGT -3′	5′- TGGCCCAACATGACTAAGGG -3′
β-actin	5′- ACTGCCGCATCCTCTTCCTC – 3′	5′- ACTCCTGCTTGCTGATCCACAT -3′

**Table 2:** Semi-quantitative assessment of the severity of disease in the knee joint according to histopathological alterations.

Histopathological Alterations			Groups		
	Healthy Control	CIA	MSCs	IL-4	MSCs+ IL-4
Hyperplasia of synovial lining layer	0	3	0	0	0
Infiltration of leukocytes into synovial membrane	0	3	1	1	0
Pannus formation	0	2	0	1	0
Necrosis/erosion of cartilage	0	3	0	1	0
**Final Arthritis Score**	0	11	1	3	0

## Supplementary Materials

The following are available online at https://www.mdpi.com/2073-4409/8/8/823/s1, Figure S1: Histopathological changes and severity of arthritis in current CIA murine model: (a) Severe; (b) Moderate; and (c) Mild CIA in mice.
