# Supplementary material for: Combination Therapy of Mesenchymal Stromal Cells and Interleukin-4 Attenuates Rheumatoid Arthritis in a Collagen-Induced Murine Model
Source: Cells. 2019 Aug 3;8(8):823. doi: 10.3390/cells8080823 (PMC6721641; doi:10.3390/cells8080823)
Supplement: Supplementary file 1 [file cells-08-00823-s001.pdf]

Supplementary Materials

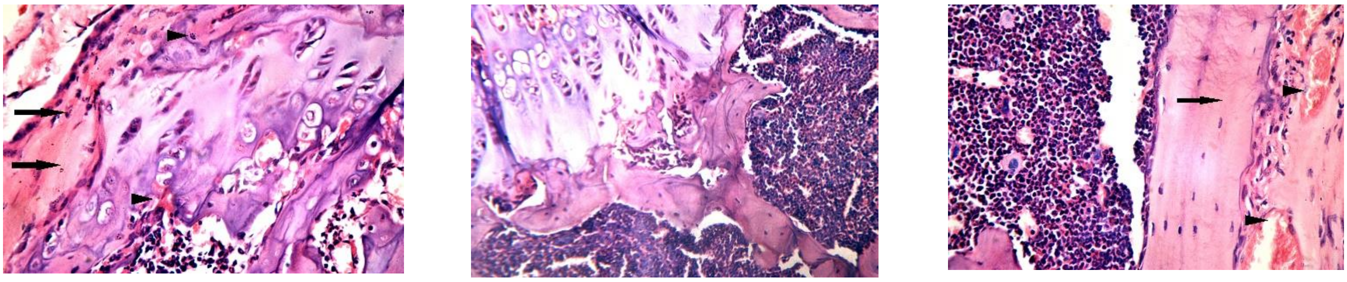

(a) (b) (c)

Figure S1. Histopathological changes and severity of arthritis in current CIA murine model: (**a**) Severe; (**b**) Moderate; and (**c**) Mild CIA in mice.
